# Diagnostic accuracy of thoracic CT to differentiate transudative from exudative pleural effusion prior to thoracentesis

**DOI:** 10.1186/s12931-024-02681-w

**Published:** 2024-01-23

**Authors:** Yan Zhang, Yang Zhang, Wei Wang, Xiaoyu Feng, Jiahuan Guo, Bo Chen, Fuyun Zhang, Huanhuan Wang, Mengnan Fan, Yingwei Zhu, Yuxia Sun, Tongsheng Wang, Yimin Mao, Pengfei Gao

**Affiliations:** 1 Department of General Medicine, The First Affiliated Hospital, College of Clinical Medicine, Henan University of Science and Technology, Luoyang, Henan China; 2 Department of Respiratory and Critical Care Medicine, The First Affiliated Hospital of Zhengzhou University, Zhengzhou, Henan China; 3 Department of Respiratory and Critical Care Medicine, The First Affiliated Hospital, College of Clinical Medicine, Henan University of Science and Technology, 24 Jing Hua Road, Luoyang, 471003 Henan China; 4 Department of Medical Record, The First Affiliated Hospital, College of Clinical Medicine, Henan University of Science and Technology, Luoyang, Henan China

**Keywords:** Pleural effusion, Thoracic CT, Diagnosis, Exudate, Transudate

## Abstract

**Background:**

Computed tomography (CT) scan is commonly performed for pleural effusion diagnostis in the clinic. However, there are limited data assessing the accuracy of thoracic CT for the separation of transudative from exudative effusions. The study aimed to determine the diagnostic value of thoracic CT in distinguishing transudates from exudates in patients with pleural effusion.

**Methods:**

This is a two-center retrospective analysis of patients with pleural effusion, a total of 209 patients were included from The First Affiliated Hospital of Henan University of Science and Technology as the derivation cohort (Luoyang cohort), and 195 patients from the First Affiliated Hospital of Zhengzhou University as the validation cohort (Zhengzhou cohort). Patients who underwent thoracic CT scan followed by diagnostic thoracentesis were enrolled. The optimal cut-points of CT value in pleural fluid (PF) and PF to blood CT value ratio for predicting a transudative vs. exudative pleural effusions were determined in the derivation cohort and further verified in the validation cohort.

**Results:**

In the Derivation (Luoyang) cohort, patients with exudates had significantly higher CT value [13.01 (10.01–16.11) vs. 4.89 (2.31–9.83) HU] and PF to blood CT value ratio [0.37 (0.27–0.53) vs. 0.16 (0.07–0.26)] than those with transudates. With a cut-off value of 10.81 HU, the area under the curve (AUC), sensitivity, specificity, positive predictive value (PPV) and negative predictive value (NPV) of CT value were 0.85, 88.89%, 68.90%, 43.96%, and 95.76%, respectively. The optimum cut-value for PF to blood CT value ratio was 0.27 with AUC of 0.86, yielding a sensitivity of 61.11%, specificity of 86.36%, PPV of 78.57%, and NPV of 73.08%. These were further verified in the Validation (Zhengzhou) cohort.

**Conclusions:**

CT value and PF to blood CT value ratio showed good differential abilities in predicting transudates from exudates, which may help to avoid unnecessary thoracentesis.

**Supplementary Information:**

The online version contains supplementary material available at 10.1186/s12931-024-02681-w.

## Background

Pleural effusion is the pathologic accumulation of fluid in the pleural cavity, a closed space between the parietal and visceral pleura, due to numerous pathologic conditions with increased production (and/or a reduction in reabsorption) of pleural fluid (PF), including heart failure, lung infection, malignant tumors, collagen vascular disease, trauma, and so forth [1]. The first step in the evaluation of PF is to distinguish exudative from transudative effusion. For the past several decades, exudative effusion has been differentiated from transudative effusion using Light’s criteria, which requires detection of protein and lactate dehydrogenase (LDH) levels in the PF through thoracentesis [2]. The goal of the initial thoracentesis is to determine the presence of exudate and alleviate dyspnea (if present). If the etiology of the pleural effusion favors leakage and there is supportive clinical history, such as heart failure, nephrotic syndrome or liver cirrhosis, thoracentesis may be postponed until it is clear whether the pleural effusion can be treated concurrently with direct treatment of the underlying disease [3]. Therefore, accurate prediction of pleural effusion diagnosis before thoracentesis will have great influence on treatment method.

Morphologic features evaluated by computed tomography (CT), such as pleural thickening, pleural nodules, loculation and effusion density, and extrapleural fat tissue thickness, may aid in the differential diagnosis of pleural effusions [4–7]. Accumulating evidence have shown that anemia can be detected on unenhanced CT of the thorax, based on density change of blood reflected by quantitative analysis of CT values [8–11]. Moreover, an obvious linear correlation of hemoglobin concentration and CT attenuation has also been clearly demonstrated [10, 11]. Accordingly, it is reasonable to speculate that density differences between exudative and transudative effusions can be detected by CT value measurement. Indeed, several studies have evaluated the efficacy of CT value to differentiate transudative from exudative pleural effusion [5, 12–14]. Nevertheless, results from these studies are inconsistent or contradictory. Thus, in current study, we aim to investigate the diagnostic value of CT in distinguishing exudative and transudative effusions. Furthermore, we also assessed the strength of correlation between CT value and patients’ protein and LDH level in PF.

## Methods

### Study design and population

This retrospective study was approved by the ethics committee of the First Affiliated Hospital of Henan University of Science and Technology (2023-03-K0027), and the ethics review board of the First Affiliated Hospital of Zhengzhou University agreed to participate. Due to the retrospective study design, the requirement for written informed consent was waived. No identifiable patient information or patient images were included in this manuscript.

**Fig. 1:**
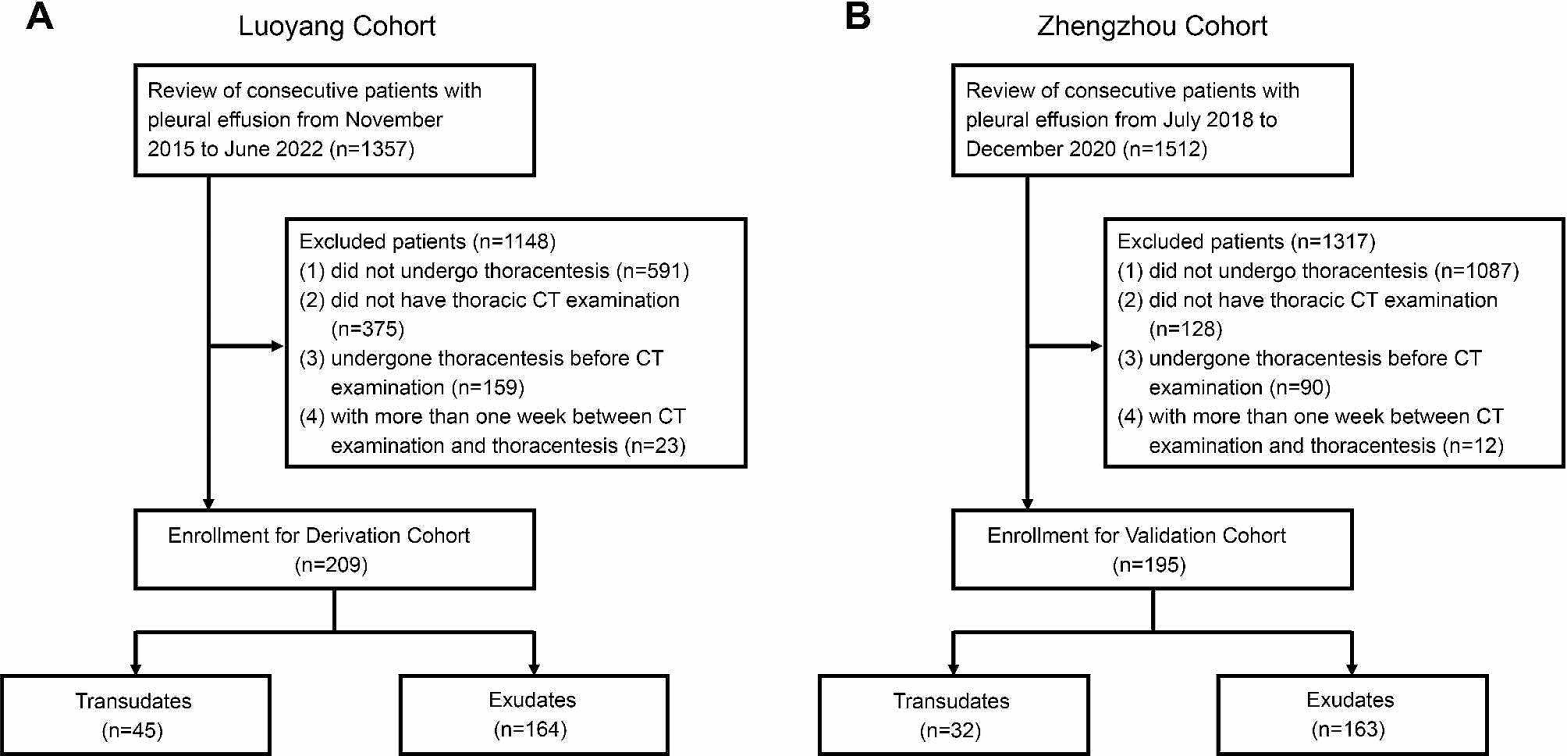
Flow chart of patient enrolment in Luoyang Cohort (**A**) and Zhengzhou Cohort (**B**)

We initially screened all of 1357 consecutive patients with a diagnosis of pleural effusion admitted to the Department of Respiratory and Critical Care Medicine between October 2015 to June 2022, from the electronic medical record of the First Affiliated Hospital of Henan University of Science (Luoyang cohort). Finally, 209 patients were included in this study, and 1148 were excluded based on the following reasons: (1) did not undergo thoracentesis (*n* = 591); (2) did not have thoracic CT examination (*n* = 375); (3) undergone thoracentesis before CT examination (159); (4) with more than one week between CT examination and thoracentesis (*n* = 23) (Fig. 1A). Luoyang cohort was used to evaluated the diagnostic capability of thoracic CT to distinguish transudative from exudative pleural effusion. Next, we validated this method using an independent cohort of 195 patients with pleural effusions from Department of Respiratory and Critical Care Medicine of the First Affiliated Hospital of Zhengzhou University (Zhengzhou cohort), and the enrollment chart was provided as: a total of 1512 consecutive patients with pleural effusion were screened, and 1317 were excluded based on the following reasons: (1) did not undergo thoracentesis (*n* = 1087); (2) did not have thoracic CT examination (*n* = 128); (3) undergone thoracentesis before CT examination (90); (4) with more than one week between CT examination and thoracentesis (*n* = 12) (Fig. 1B).

### Diagnostic criteria

According to Light’s criteria, pleural effusions were classified as either transudate or exudative [2]. Pleural effusions were classified as exudative when any one of the following findings was present: (1) PF to serum protein ratio > 0.5, (2) PF to serum LDH ratio > 0.6, or (3) PF LDH higher than two thirds of the upper limit of normal in serum; otherwise, the effusion was classified as transudate.

### Thoracic CT scan and data acquisition

**Fig. 2:**
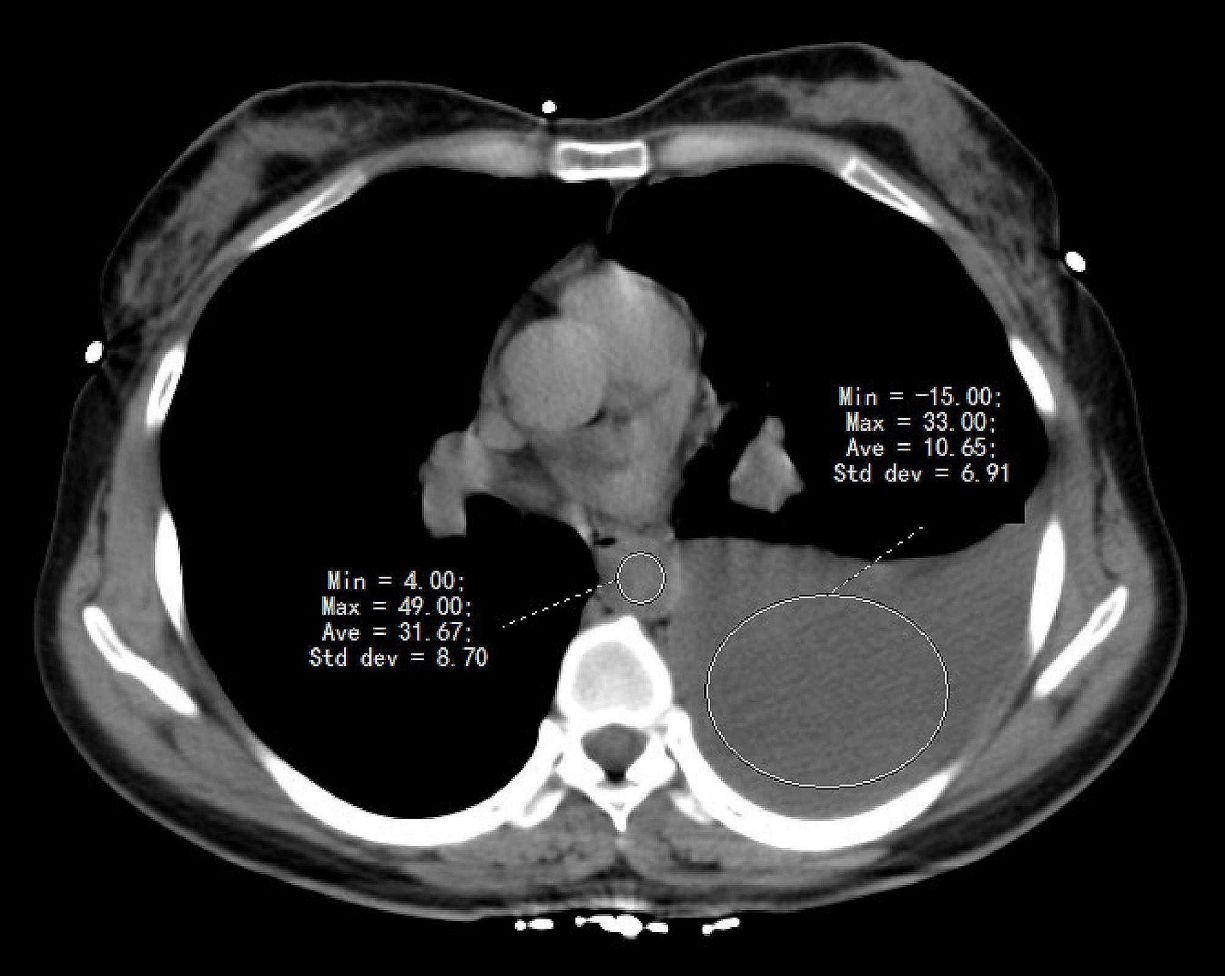
ROIs were placed on the greatest amount of effusion for the measurement of CT values. Blood pool HU values of aorta cavity were also obtained in the same manner. ROIs, regions of interest

All CT imaging studies were volume scans performed with a 64-multiple detector CT scanner (Lightspeed VCT; GE Healthcare, Milwaukee, WI, USA) in Luoyang Cohort, and with a 128-multiple detector CT scanner (NeuViz Glory; Neusoft Medical Systems, Shenyang, China) or 256-multiple detector CT scanner (NeuViz Epoch; Neusoft Medical Systems, Shenyang, China) in Zhengzhou Cohort, using the following scan parameters: tube voltage 120 kV, automatic tube current setting, and section thickness of 5 mm. All CT-scans were performed without intravenous contrast. Regions of interest (ROIs) were selected on the greatest amount of effusion on each slice of the three slices used for the measurement of Hounsfield Unit (HU) values. HU values were measured three times by investigators who were blinded to the clinical characteristics and laboratory findings, then the mean of the three HU values was calculated. Blood pool HU values of aorta cavity were also obtained in the same manner (Fig. 2).

### Statistical analyses

All distributions passed tests for normality. Continuous variables with normal distribution, described as mean ± standard deviations, were compared by independent group t test. For non-normally distributed data, results were presented as medians with interquartile ranges, and Mann-Whitney test was used. Fisher exact test was used for analysis of categorical data. Pearson correlation was used to analyze association between normally distributed variables. For nonnormally distributed data, we analyzed correlation using Spearman rank order correlation. Receiver operating characteristic (ROC) curve analysis was performed, and areas under the curves (AUCs) were calculated to evaluate the diagnostic efficiency of the CT value and PF to serum CT value ratio, including sensitivity, specificity, positive predictive value (PPV) and negative predictive value (NPV). We attempted to establish the optimal cut-off values for CT value and PF to blood CT value ratio using Youden index [15]. In the validation set, we tested the universality of the cut-off values for distinguishing exudative from transudative effusion. Measurement agreement was tested using inter-class correlation coefficient values. All statistical analyses were performed with GraphPad Prism 6 (GraphPad Software, San Diego, CA, USA) and SPSS version 22 (SPSS Inc., Chicago, IL, USA). *P* < 0.05 was considered statistically significant.

## Results

### Study population

Baseline characteristics of 209 patients from derivation cohort and 195 patients from validation cohort were shown in Table 1. There was no significant difference between exudates and transudates in terms of sex distribution. Patients with transudative pleural effusions were older than those with exudates in Luoyang Cohort, which was not observed in Zhengzhou Cohort, this might be attributed to the differences in disease spectrum. As expected, protein and LDH levels were significantly higher in patients with exudative pleural effusions, when comparing to those with transudative, in both Luoyang and Zhengzhou Cohort.

**Table 1:** General characteristics of Luoyang and Zhengzhou Cohort

Characteristics	Luoyang Cohort (Derivation Cohort)			Zhengzhou Cohort (Validation Cohort)		
	transudates	exudates	p value	transudates	exudates	p value
Number	45	164	NA	32	163	NA
Sex (F/M)	16/29	52/102	0.859	17/15	44/119	0.006
Age (y)	67.50 (32.00–77.00)	57.00 (14.00–70.00)	0.012	65.50 (53.25–74.75)	65.00 (55.00–72.00)	0.697
Protein (g/L)	19.45 ± 5.88	41.04 ± 10.04	< 0.001	20.56 ± 6.95	42.08 ± 10.01	< 0.001
LDH (U/L)	76.00 (67.50–100.00)	292.50 (168.80-535.50)	< 0.001	112.50 (82.00-132.00)	320.50 (179.00-533.30)	< 0.001
Glucose (mmol/L)	7.56 (6.41–9.55)	6.60 (5.19–7.91)	< 0.001	7.30 (5.46–9.44)	6.45 (5.29–8.16)	0.063

Data are presented as mean ± SD or median (interquartile range). LDH, lactate dehydrogenase; NA, not applicable

### CT value measurement agreement

To evaluate the CT value measurement agreement, an investigator measured all CT values of pleural effusion and aorta cavity, while another investigator repeated the measurement in Luoyang Cohort. The intra-class correlation coefficient values were 0.968 for pleural effusion, and 0.955 for aortic cavity, respectively, indicating perfect repeatability of CT value measurements (Table 2).

**Table 2:** Inter-observer agreement for CT value measurement

	Intra-class correlation coefficient	95% confidence interval
Pleural effusion	0.968	0.958–0.976
Aortic cavity	0.955	0.940–0.965

### CT value and PF to blood CT value ratio in transudates and exudates

**Fig. 3:**
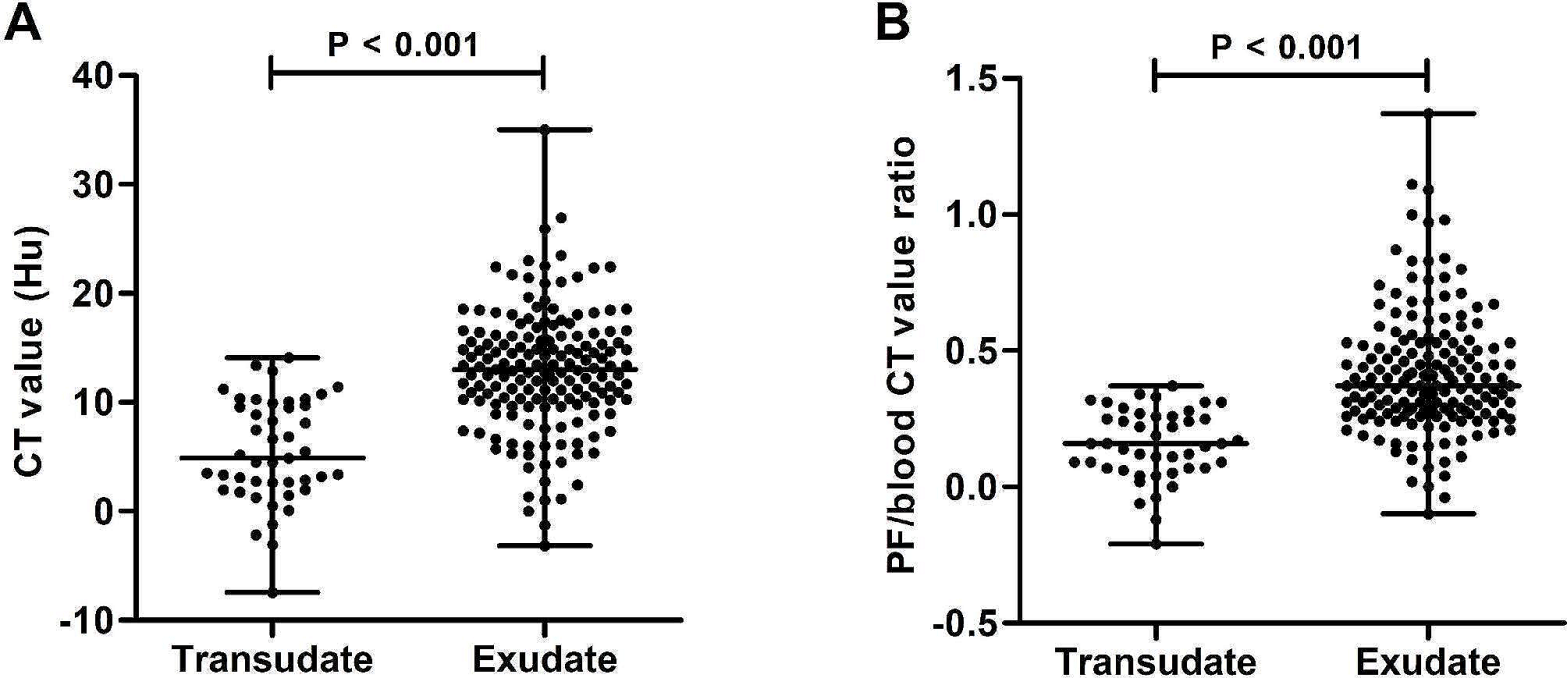
Comparison of CT value in PF (**A**) and PF/blood value ratio (**B**) in patients with transudative and exudative effusion in Luoyang Cohort. CT, computed tomography; PF, pleural fluid

In Luoyang Cohort, the median CT value [13.01 (10.01–16.11) HU] of the exudates were significantly higher than those of the transudates [4.89 (2.31–9.83) HU] (*P* < 0.001) (Fig. 3A). Moreover, PF to blood CT value ratio in patients with exudative pleural effusions [0.37 (0.27–0.53)] were also significantly elevated compared to patients with transudative effusions [0.16 (0.07–0.26)] (*P* < 0.001) (Fig. 3B). Similar results were also observed in Zhengzhou Cohort, with significantly higher CT value [11.96 (8.58–15.51) vs. 6.08 (2.49–9.93) HU] (Figure S1A) and PF to blood CT value ratio [0.31 (0.26–0.39) vs. 0.18 (0.08–0.26)] (Figure S1B) in patients with exudates than those with transudates.

### Correlation analysis of CT attenuation and protein level in plural effusion

**Fig. 4:**
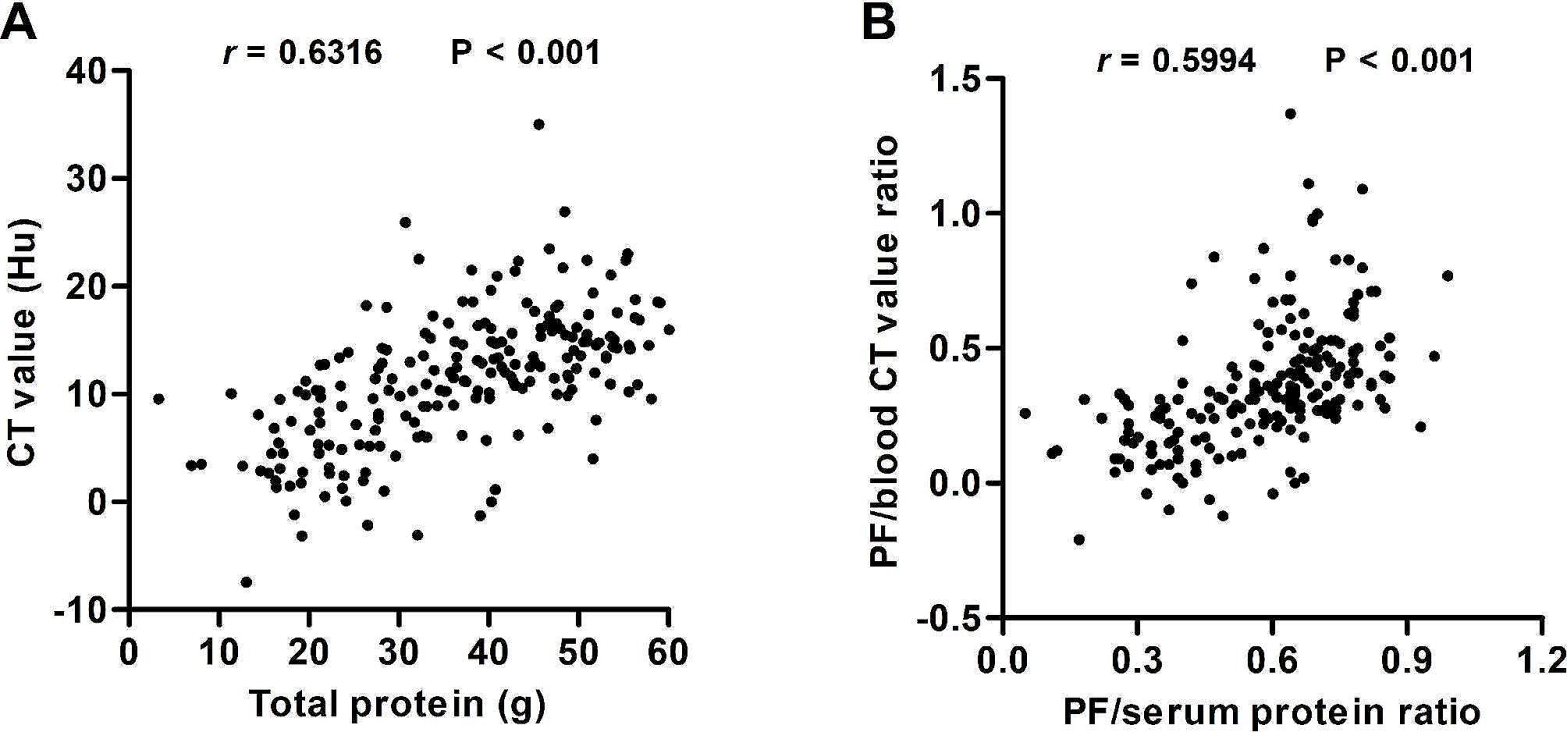
Correlation analysis of total protein level and CT value in plural effusion (**A**), and PF/serum protein ratio and PF/blood CT value ratio (**B**) in Luoyang Cohort. CT, computed tomography; PF, pleural fluid

Relative analysis revealed positive correlation of CT values with protein levels (*r* = 0.63, *P* < 0.001) in pleural effusions in Luoyang Cohort (Fig. 4A). In addition, PF to blood CT value ratio were also found to be positively correlated with PF/serum protein ratio (*r* = 0.60, *P* < 0.001) (Fig. 4B). Importantly, we were able to recapitulate such positive correlations in Zhengzhou Cohort (Figure S2).

### Diagnostic performance of CT value and PF to blood CT value ratio for distinguishing transudative from exudative pleural effusion

**Fig. 5:**
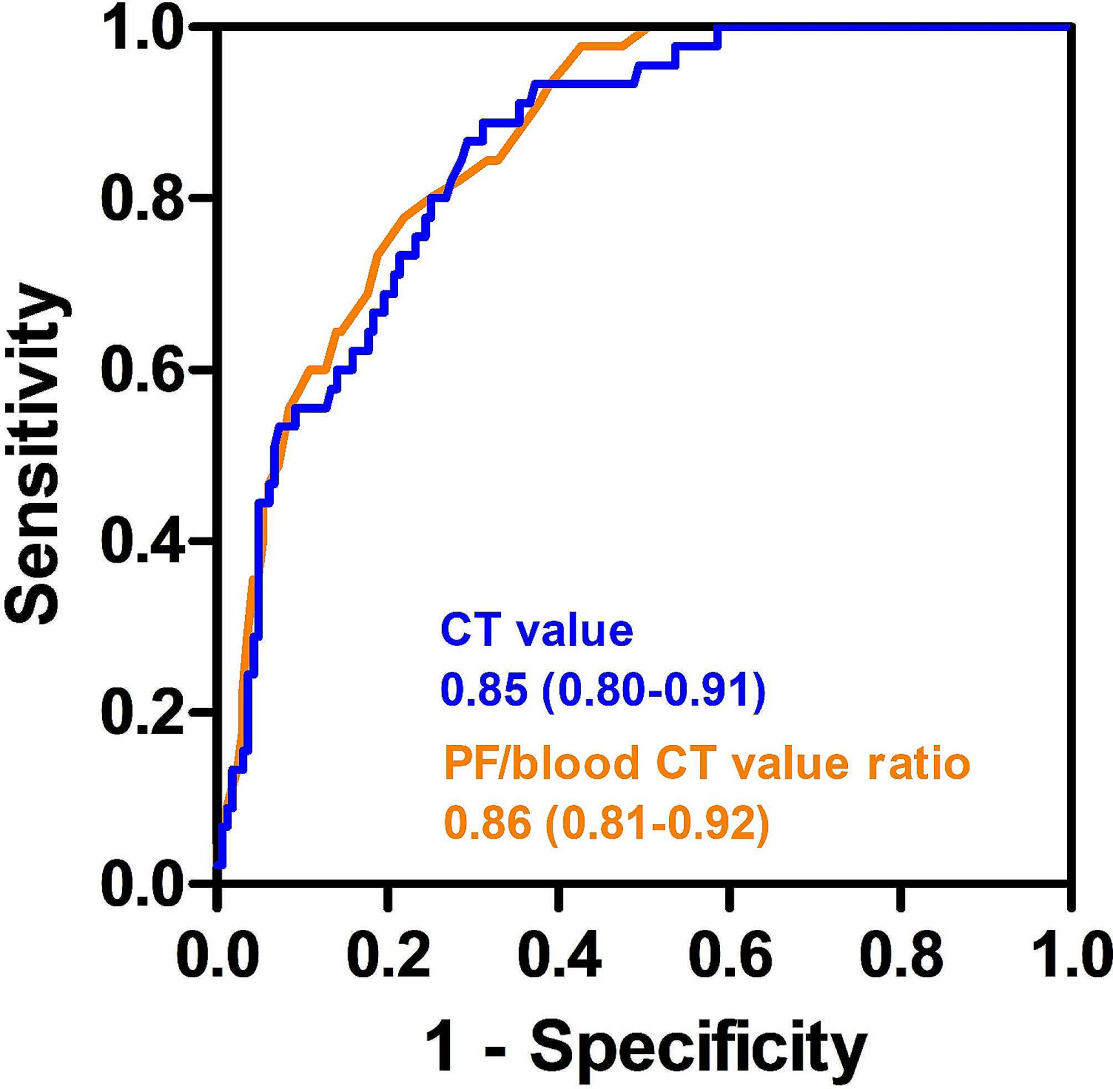
Receiver operating characteristic curve analysis of CT value and PF/blood CT value ratio for distinguishing patients with transudative pleural effusion from those with exudative effusion in Luoyang Cohort. CT, computed tomography; PF, pleural fluid

The ROC curves derived from Luoyang Cohort demonstrate the diagnostic capability of CT value and PF to blood CT value ratio to distinguish transudative from exudative pleural effusion, as shown in Fig. 5. Youden index of CT value was 0.58. With a cut-off value of 10.81 HU in Luoyang Cohort, the AUC of CT value to differentiate transudative from exudative pleural effusions was 0.85 (95% CI, 0.80 to 0.91) (Fig. 5), with sensitivity of 88.89%, specificity of 68.90%, PPV of 43.96%, and NPV of 95.76%, respectively (Table 3); whereas the AUC for PF to blood CT value ratio was 0.86 (95% CI, 0.81 to 0.92) (Fig. 5). When the cut-off value is set to 0.27, with Youden index of 0.56, the diagnostic performance of PF to blood CT value ratio reaches 77.78% sensitivity, with specificity, PPV, and NPV being 78.05%, 49.30%, and 92.75%, respectively (Table 3). Using cut-off values of the CT value and PF to blood CT value ratio obtained from Luoyang Cohort, we performed a separate retrospective validation study (Zhengzhou Cohort). As the results shown in Table 3, our method shows a sensitivity of 84.38%, a specificity of 60.74%, a PPV of 29.67%, and a NPV of 95.19% for distinguishing transudative from exudative pleural effusion with CT value. Furthermore, the sensitivity, specificity, PPV, and NPV of PF to blood CT value ratio were 78.13%, 74.85%, 37.88%, and 94.57%, respectively (Table 3).

**Table 3:** Diagnostic performance of CT value and pleural fluid/blood CT value ratio in differentiating between patients with transtive and exuative pleural effusion

	Cut-off value	Sensitivity (%)	Specificity (%)	PPV (%)	NPV (%)
Luoyang Cohort	CT value, 10.81 HU	88.89	68.90	43.96	95.76
	Pleural fluid/blood CT value ratio, 0.27	77.78	78.05	49.30	92.75
Zhengzhou Cohort	CT value, 10.81 HU	84.38	60.74	29.67	95.19
	Pleural fluid/blood CT value ratio, 0.27	78.13	74.85	37.88	94.57

Cut-off values were established using Youden index in Luoyang Cohort.

PPV, positive predictive value; NPV, negative predictive value

## Discussion

This two-center retrospective study confirmed the difference in CT values between transudative and exudative effusions, and demonstrated the high diagnostic accuracy of CT values. Moreover, we proposed that a new relative index, PF to blood CT value ratio, can be used to distinguish exudates from transudates with better universality. Importantly, the diagnostic application of CT value and PF to blood CT value ratio evaluated in Luoyang Cohort was further validated using a separate cohort of patients (Zhengzhou Cohort). The strong positive correlation between CT value and protein level in pleural effusion is fundamental for the differential diagnostic performance. Our findings may reduce the incidences of unnecessary thoracentesis in patients with transudative pleural effusion.

The application of CT scan is well established in the diagnostic process for pleural effusion treatment. Previous studies have suggested that certain CT scan features, namely pleural thickening, pleural nodularity and loculation, are highly suggestive of exudative pleural effusion with an excellent specificity [4–7, 12, 13, 16]. Nevertheless, the absence of these features in a substantial proportion of exudative pleural effusions will reduce the sensitivity significantly. Therefore, it is necessary to measure the specific CT value to improve differential diagnosis capability. However, there are very few high-quality evidence on diagnostic accuracy of CT attenuation value to distinguish transudative from exudative pleural effusion. Kiran et al. [14], Kadihan et al. [13], and Neşat et al. [5] independently reported that the mean CT attenuation value of the exudates was significantly higher than those of the transudates, but the diagnostic accuracy was suboptimal. However, these studies show dampening limitations: such as (1) study size was small, (2) did not show rigorous workflow of patient enrollment, and (3) most importantly, did not validate their findings using a separate independent cohort. Our study addressed all these limitations by utilizing expanded sample size with strict execution of the enrollment criteria, and further testing our findings with an external validation patient cohort.

In contrast, another study reported no significant difference between transudative and exudative PF when evaluating the CT values of 100 patients with pleural effusion [12]. This might due to the fact that they collected data from four different CT scanners [12]. It is also worthy mentioning that the detection values of CT attenuation in pleural effusion were greatly different among various studies [5, 12–14]. Such large discrepancy might be attributed to the diverse parameters used for different CT scanners, and the CT value of PF was inconsistent at different medical institutions. Meanwhile, relative ratios such as PF to serum protein and LDH ratio, are key components of Light’s criteria [2]. Our study used the relative index of PF and blood CT value ratio for the first time, which can avoid inconsistencies in measurement results under different clinical situations to the greatest extent; the PF to blood CT value ratio was proven to have similar diagnostic efficacy in a single center, but demonstrated better universality in an independent cohort.

Consistent with previous findings, our data showed positive correlation between CT values and laboratory markers of Light’s criteria [14]. Moreover, we proposed and confirmed fort the first time that the ratio of PF to blood CT value was strongly correlated with the ratio of PF to serum protein. Similarly, the CT value of whole blood was linearly related to hemoglobin levels [10, 11]. These data provided a theoretical explanation for the differential diagnosis of pleural effusion and the detection of anemia through CT value measurement.

There is an overlap in CT values for transudates and exudates in a considerable part of effusions, whether in current or previous studies [5, 12–14]. This is partly attributed to the presence of multiple underlying causes in approximately one-third of pleural effusion cases, leading to potential coexistence of both transudative and exudative mechanisms in effusion formation [17]. Infections are common in patients with hypoproteinemia, cirrhosis, or nephrotic syndrome due to impaired immune function; and the incidence of multifactorial pleural effusion is rapidly growing with higher incidences of cardiovascular and malignant disease [18]. Indeed, Light’s criteria itself, as a binary classification system dividing effusion into transudate and exudate while presuming a single disease process driving fluid accumulation, was not originally designed to recognize concurrent etiologies [19]. Similarly, the approach of CT value measurement also had innate limitation to detect pleural effusions with mixed mechanisms, accompanied by relatively low positive predictive value. Nonetheless, CT value > 15 HU or PF to blood CT value ratio > 0.4 strongly suggests the presence of exudate. Conversely, transudate should be carefully considered when the CT value was < 5 HU, or PF to blood CT value ratio < 0.15. In general, through the utilization of quantitative analysis of CT attenuation in conjunction with distinctive imaging indicators, thoracic CT scan could effectively differentiate transudative and exudative pleural effusion with a reasonably high degree of accuracy.

Although the study investigators were blinded to the clinical information when they measured CT values, these investigators were the treating physicians for some of these cases, this may potentially introduce recall bias. Second, not all CT scans were performed by the same machine, especially in Zhengzhou Cohort, which might have caused a measurement bias and limit the generalizability. To address these limitations, PF to blood CT value ratio was proposed in this study which can reduce inconsistent measurements from different CT scanners. Third, it is difficult to completely eliminate subjectivity in the acquisition of CT values, but inter-observer variability analysis showed good reproducibility. Fourth, as previously described, there were some CT scan features that may be specific for differential diagnosis of pleural effusion, but these were not specifically analyzed in our research. Lastly, due to the nature of our retrospective study, future studies are warranted to prospectively validate our findings.

## Conclusions

our study demonstrated the feasibility of using CT value to distinguish exudates from transudates with high accuracy. PF to blood CT value ratio was also confirmed to show fair performance to predict transudative vs. exudative pleural effusion. These findings may aid physicians in making preliminary assessments of pleural effusion prior to thoracentesis, thereby minimizing unnecessary thoracentesis.

### Electronic supplementary material

Below is the link to the electronic supplementary material.

Supplementary Material 1

Supplementary Material 2

Supplementary Material 3

## Data Availability

The full data and materials of the current study are not publicly available due to limitations of ethical approval involving the patient data but are available from the corresponding author on reasonable request.

## Abbreviations

LDH: Lactate dehydrogenase
CT: Computed tomography
HU: Hounsfield Unit
ROC: Receiver operating characteristic
AUC: Areas under the curves
PPV: Positive predictive value
NPV: Negative predictive value
PF: Pleural fluid
