# Supplementary figures and images for: Diagnostic accuracy of thoracic CT to differentiate transudative from exudative pleural effusion prior to thoracentesis
Source: Respir Res. 2024 Jan 23;25:53. doi: 10.1186/s12931-024-02681-w (PMC10807107; doi:10.1186/s12931-024-02681-w)

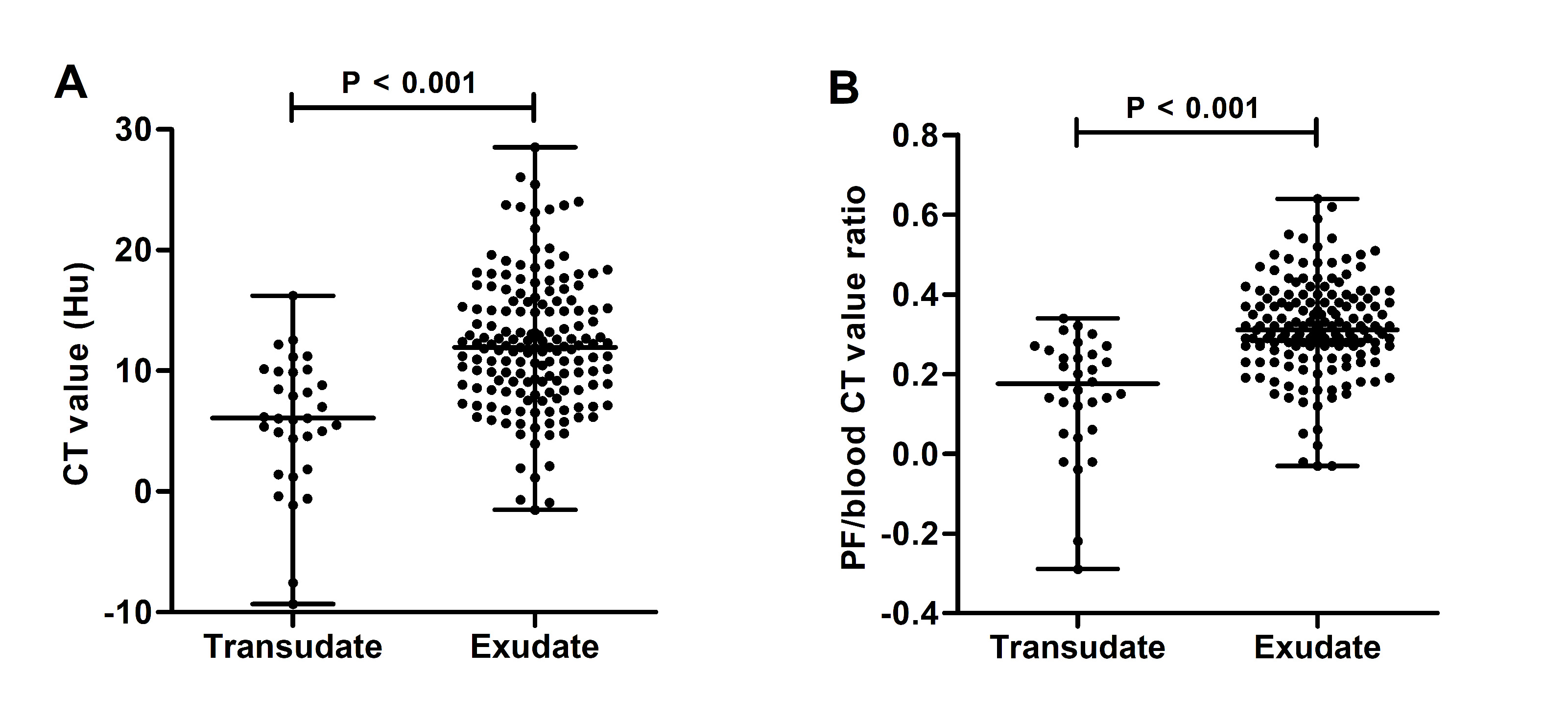

Supplement: Supplementary file 1 — Supplementary Material 1 [file 12931_2024_2681_MOESM1_ESM.jpg]

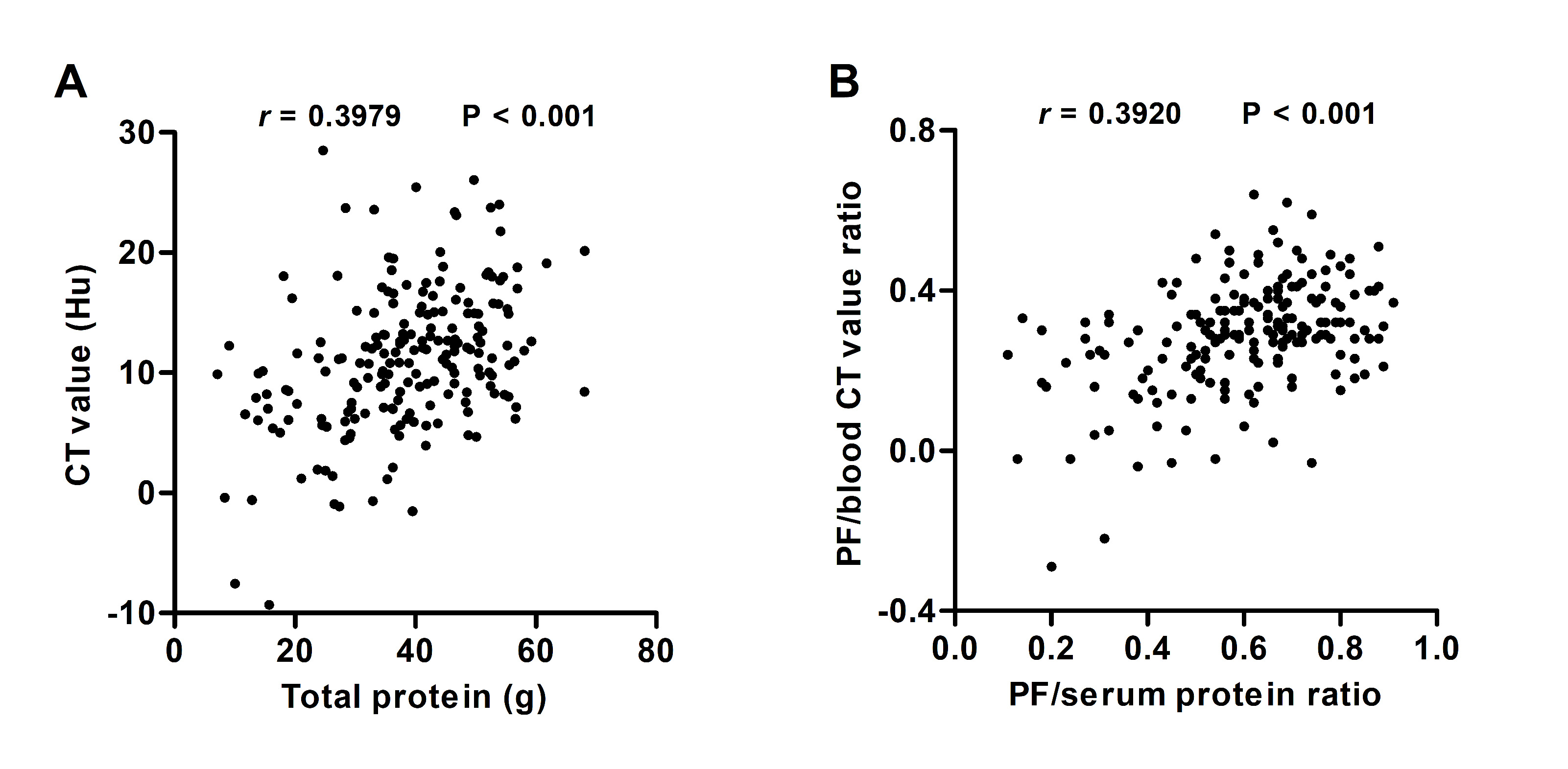

Supplement: Supplementary file 2 — Supplementary Material 2 [file 12931_2024_2681_MOESM2_ESM.jpg]

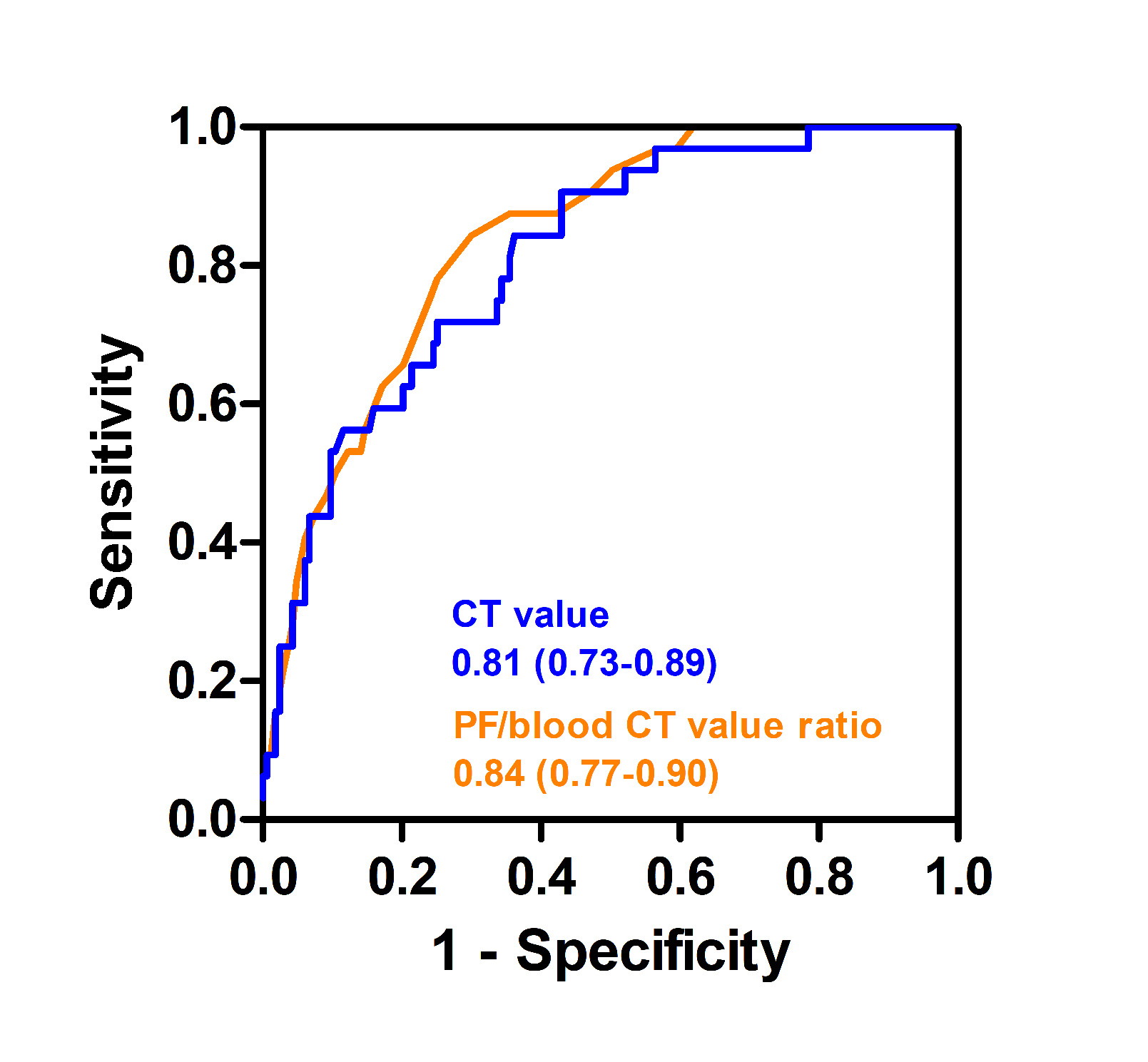

Supplement: Supplementary file 3 — Supplementary Material 3 [file 12931_2024_2681_MOESM3_ESM.jpg]
